# Case of a Young Male Recruit With Chest Pain Leading to Entry-Level Separation and Career Termination

**DOI:** 10.7759/cureus.76388

**Published:** 2024-12-25

**Authors:** Syed M Naqvi, Amin Ur Rehman Nadeem

**Affiliations:** 1 Pulmonary Disease, Chicago Medical School, Rosalind Franklin University of Medicine and Science, North Chicago, USA; 2 Critical Care, Captain James A. Lovell Federal Health Care Center, Rosalind Franklin University of Medicine and Science, North Chicago, USA

**Keywords:** acute coronary syndrome, atypical chest pain, chest pain, myocardial bridging, young male

## Abstract

This is a case of a young, 20-year-old, male Navy recruit who was admitted to our healthcare facility with intermittent atypical chest pain and limiting exertional symptoms and was diagnosed with myocardial bridging (MB) as the most likely etiology of his chest after the complete cardiac workup, leading to his career limitations due to potential risks. Our patient presented with atypical chest pain and limiting exertional symptoms. Chest pain was non-radiating. His family history was positive for myocardial infarction on his mother's side under the age of 40 but negative for tobacco use, family history of other cardiac anomalies, or recent illness. Vitals and initial labs were within normal limits. Chest X-ray showed no acute findings. The electrocardiogram (ECG) was noted for early repolarization and biphasic T waves in leads V2 and V3. Acute coronary syndrome (ACS) was ruled out. His transthoracic echocardiography (TTE) was normal. The cardiac stress test was negative for any reversible ischemic changes. The coronary computed tomography angiogram (CCTA) confirmed the diagnosis of symptomatic MB. The patient was started on metoprolol, and his chest pain improved. His follow-up ECG showed a resolution of T-wave changes. Based on further recommendations from cardiology, the patient had undergone entry-level separation from the Navy because of symptomatic MB. Our case emphasizes the need for awareness of this rare cause of non-atherosclerotic coronary ischemia in young patients presenting with chest pain who do not fit the picture of atherosclerotic heart disease. Therefore, timely recognition of MB in these young patients by the healthcare provider by ruling out ACS and earlier risk assessment by performing transthoracic TTE and CCTA, if indicated, is crucial and can prevent any significant events by prompt intervention and management.

## Introduction

Myocardial bridging (MB) is a congenital anomaly in which an epicardial coronary artery travels abnormally within the myocardium, called bridging, and is compressed during the ventricle systole causing symptoms in the patients. MB is usually a benign condition but can manifest as a non-atherosclerotic cause of acute coronary syndrome (ACS), arrhythmias, transient ventricular dysfunction, or sudden cardiac arrest [1]. Therefore, MB diagnosis is crucial, particularly for military recruits, as it has career implications.

We are reporting a case of a young, 20-year-old, male military (Navy) recruit who came with intermittent atypical chest pain and limiting exertional symptoms and was found to have MB as the most likely etiology of his chest pain after the complete cardiac workup. Clinicians should keep MB in their differentials as a cause of non-atherosclerotic coronary ischemia in young adults who are required to perform vigorous training.

## Case presentation

This was a 20-year-old male military (Navy) recruit admitted to the medical floor at our healthcare facility with a chief complaint of intermittent atypical chest pain and limiting exertional symptoms. This was the second time he visited the emergency room with similar symptoms.

During the first visit about a week ago, he was evaluated in the emergency room for similar complaints, and ACS was ruled out. He also had transthoracic echocardiography (TTE) with Optison contrast performed; the impression was normal left ventricular (LV) systolic and diastolic function; LV ejection fraction was 55%-60%; and there were no regional wall motion abnormalities, normal right ventricular size and systolic function, no significant valvular abnormalities, and inadequate TR jet for estimation of pulmonary artery systolic pressure. He was discharged back to the Navy base during that visit.

This time, he described the chest pain as “pressure-like” and as if he had a “strain” mostly on exertion. Chest pain was intermittent and non-radiating. He denied palpitations, shortness of breath, dizziness, nausea, vomiting, pre-syncope, or syncope. He denies any recent viral illness. He reported a positive family history of myocardial infarction in his mother under the age of 40 but denied any personal or family history of other cardiac anomalies. He denied caffeine, energy drinks alcohol, tobacco, or illicit drug use.

Physical examination including cardiac exam was unremarkable without murmur, rub, or gallop. His vitals were normal and are included in Table 1 except for mildly elevated blood pressure on initial presentation which was likely due to his chest pain during the initial presentation which subsequently came back to normal and remained within the normal range during the entire hospital stay. His labs were mostly within the normal range and are included in Table 2 except for the unexplained mild elevation in the levels of C-reactive protein and erythrocyte sedimentation rate.

**Table 1:** Initial vitals on presentation

Vital signs	Patient’s value	Normal range
Temperature	98.4^o^F	97.8-100.4^o^F
Respiratory rate	18 breaths/min	10-20 breaths/min
Heart rate	63 beats/min	60-100 beats/min
Blood pressure	141/81 mmHg	120/80 mmHg

**Table 2:** Initial labs on presentation

Labs	Patient’s value	Normal range
Hemoglobin	13.0 g/dl	13.0-17.0 g/dl
White blood cell count	10.5 K/uL	4.0-11.0 K/uL
Creatinine	0.80 mg/dl	0.67-1.17 mg/dl
Potassium	3.7 mmol/L	3.5-4.7 mmol/L
Magnesium	2.06 mg/dl	1.6-2.6 mg/dl
Blood urea nitrogen	10 mg/dl	7-21 mg/dl
High sensitivity troponin	3 ng/L	Ref: <=53 ng/L
Thyroid-stimulating hormone	1.197 uIU/ml	0.55-4.78 uIU/ml
D-dimer	284 ng/ml	0-499 ng/ml
Brain natriuretic peptide	<2 pg/ml	0-100 pg/ml
C-reactive protein	10.1 mg/L	0-10 mg/L
Erythrocyte sedimentation rate	29 mm/hr	0-15 mm/hr
International normalized ratio	1.2	0.8-1.1

**Figure 1:**
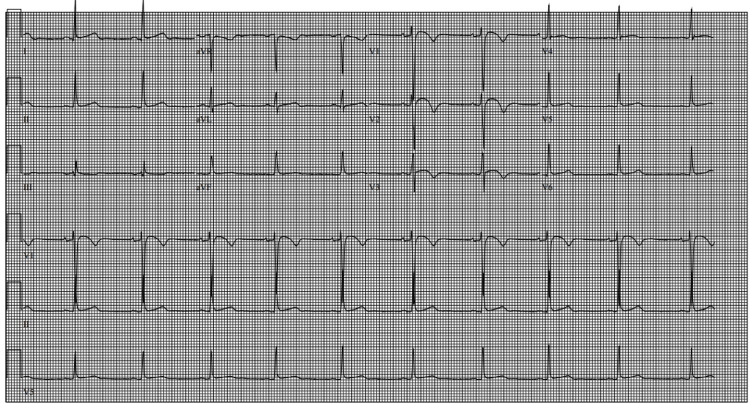
ECG showing early repolarization and biphasic T waves in leads V2 and V3 ECG: electrocardiogram

Chest X-ray showed no acute findings. The electrocardiogram (ECG) was noted for showing early repolarization and biphasic T waves in leads V2 and V3 (Figure 1).

**Figure 2:**
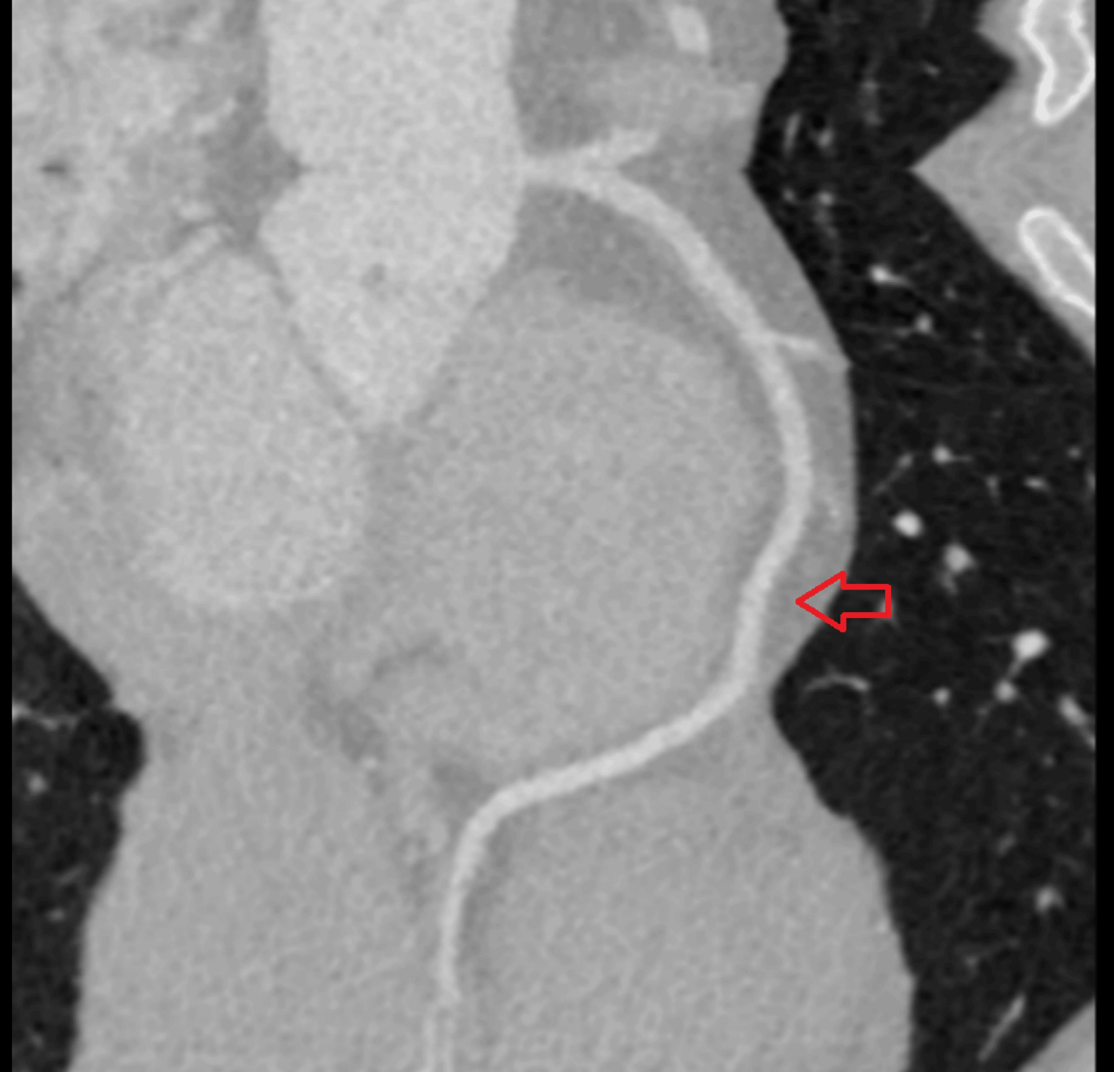
Coronary computed tomography angiogram revealing proximal left anterior descending artery myocardial bridging (shown by the red arrow)

The patient was admitted for further evaluation and cardiologic workup due to concerning ECG findings. The cardiologist recommended an exercise treadmill test (ETT) to rule out cardiac ischemia. In his cardiac stress test, performed with Bruce protocol, our patient was able to exercise for six minutes achieving a 10 metabolic equivalents score (METS), but his functional capacity was determined to be below average explaining his exertional limitations; however, results showed normal hemodynamic response without any reversible ischemic changes. Stress ECG did not show any significant ECG changes with exercise while noting that he has baseline resting changes as mentioned above. Subsequently, a coronary computed tomography angiogram (CCTA) was performed which revealed proximal left anterior descending (LAD) artery MB, likely the cause of our patient's symptoms, shown by the red arrow in Figure 2.

**Figure 3:**
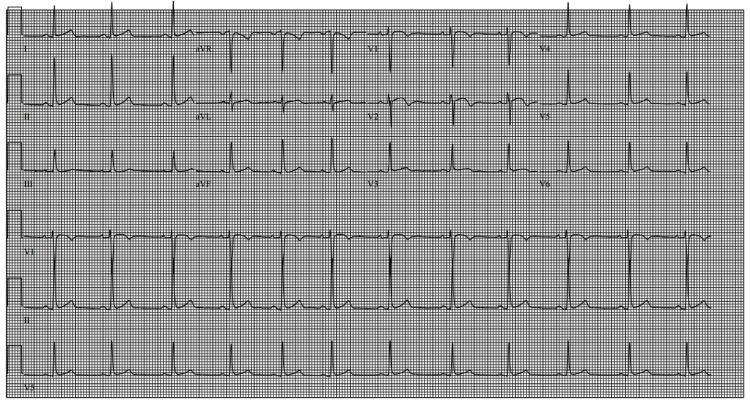
ECG showing normal sinus rhythm with sinus arrhythmia ECG: electrocardiogram

Cardiology concluded the most likely diagnosis of symptomatic MB for our patient’s intermittent chest pain and limiting exertional symptoms based on CCTA findings. The patient was started on metoprolol tartrate 25 mg twice daily, a low-dose beta-blocker which is the first-line therapy for symptomatic MB. The follow-up ECG, done a few days later, showed a resolution of early repolarization changes (Figure 3).

The patient remained hemodynamically stable and chest pain-free on the treatment regimen during the hospital stay for more than a week and was discharged back to the Navy base on the same dose of long-acting metoprolol succinate. Based on further recommendations from cardiology, the primary team recommended that the patient should undergo entry-level separation from the Navy because of symptomatic MB as this condition poses potential risks under vigorous military training signifying its importance in occupational medicine. The patient was advised to follow up with a community cardiologist for recommendations on lifestyle adjustments or physical activity limitations as well as monitoring of his symptoms and future management or intervention as deemed necessary.

## Discussion

MB is a congenital condition in which a portion of the major epicardial coronary artery travels intramurally through the muscles of the heart [1] and is compressed during the ventricle systole causing symptoms in the patients. The LAD coronary artery was exclusively involved in 67% to 98% of the cases, specifically the middle portion of the LAD coronary artery [2,3]. Coronary ischemia depends on the location, thickness/depth, and length of the myocardial bridge involving the coronary artery [3]. Many case reports have been published with different clinical manifestations and outcomes. MB often is an incidental finding and has a benign prognosis but can become symptomatic under specific conditions (such as stress or high exertion) and in our case during vigorous military training. The most likely explanation for it to become symptomatic with stress or high exertion could be related to dynamic compression of the bridged portion of the artery during systole, its association with higher heart rates, and increased sympathetic tone. In some cases, it can also present with serious life-threatening conditions such as ACS, arrhythmias, exercise-induced atrioventricular conduction blocks, transient ventricular dysfunction, or sudden cardiac arrest [4-8].

The crucial step is to differentiate between functional findings and anatomic anomalies. Various diagnostic modalities including but not limited to coronary computed tomography angiography, coronary angiography with the use of provocative testing, intracoronary Doppler imaging, or intravascular ultrasonographic imaging can be utilized to identify MB [1,8,9]. The prevalence of intramuscular coronary arteries was reported to be approximately 30% in coronary computed tomography angiography (CCTA) [9]. In further studies, only 50% of patients had a culprit LAD coronary artery lesion [10]. Furthermore, MB may lead to coronary ischemia during strenuous exercise or stress, but its ECG findings are not specific [11].

In our patient, aggravating angina symptoms were likely considered to be due to new stress as a recruit and vigorous training which increased the sympathetic tone and resulted in the symptoms which were overlooked before joining the Navy boot camp. Pharmacological treatment is the first-line management for symptomatic MB. Based on the above mechanisms for ischemia, beta-blockers, and non-dihydropyridine calcium channel blockers are preferred due to their negative inotropic and chronotropic effect [1,4]. Nitrates can cause secondary tachycardia along with reflex sympathetic stimulation and therefore, contraindicated [1,4]. Coronary artery stenting, surgical myotomy, or coronary artery bypass grafting can be pursued in cases refractory to medical management [12-13]. In cases where a coronary stent had been placed, stent fracture is noted as a recurrent complication due to MB [14]. 

Our patient was managed conservatively with medical therapy without any need for surgery. On follow-up, our patient remained chest pain-free on medical management alone. The resolution of ECG findings also indicates a good response to the medical therapy in our patient.

## Conclusions

Our case was a young male recruit whose training was terminated due to his symptomatic MB; he was placed on entry-level termination, but in doing so, he most likely was spared from the life-threatening complications. Although atherosclerotic coronary artery disease is the main cause of ACS, it is important to consider non-atherosclerotic etiologies of chest pain such as MB, but the mechanism of ischemia is tangled and poorly understood. Our case report also highlighted the importance of understanding the basic pathophysiology, different imaging choices for the diagnosis, and treatment options for this rare but challenging cardiac condition.

Timely recognition of MB by the care providers is crucial in the prevention of any ruinous events such as myocardial infarction, arrhythmias, exercise-induced atrioventricular conduction blocks, transient ventricular dysfunction, syncope, or even sudden cardiac arrest and could also prevent mortality by prompt intervention and management. In summary, our case represents the rare cause of non-atherosclerotic coronary ischemia which should be kept in the differential diagnosis in patients presenting with chest pain who do not fit the picture of atherosclerotic heart disease.
